# Difference in Volatile Aroma Components of *Stropharia rugosoannulata* under Two Cultivated Environments Investigated by SPME-GC-MS

**DOI:** 10.3390/foods12142656

**Published:** 2023-07-10

**Authors:** Yanbin Wang, Dan Wu, Yingqi Wu, Xiaoqing Tong, Yuchuan Qin, Liling Wang

**Affiliations:** 1 Zhejiang Academy of Forestry, Hangzhou 310023, China; numbsword@126.com (Y.W.); txq153@sohu.com (X.T.); hzqinchuan@126.com (Y.Q.); echo22239@163.com (L.W.); 2 National-Local Joint Engineering Laboratory of Intelligent Food Technology and Equipment, Zhejiang Key Laboratory for Agro-Food Processing, Integrated Research Base of Southern Fruit and Vegetable Preservation Technology, Zhejiang International Scientific and Technological Cooperation Base of Health Food Manufacturing and Quality Control, Fuli Institute of Food Science, College of Biosystems Engineering and Food Science, Zhejiang University, Hangzhou 310058, China; 3 Qingyuan Bureau of Natural Resources and Planning, Qingyuan 323800, China; ar-thur-tulang@163.com

**Keywords:** *Stropharia rugosoannulata*, cultivation mode, volatile aroma compounds, mushroom, gas chromatography—mass spectrometry

## Abstract

In order to study the effect of both greenhouse and forest cultivating environments on *Stropharia rugosoannulata*, its volatile aroma compounds were measured by a headspace solid phase micro extractions—gas chromatograph—mass spectrometer (SPME–GC–MS). The optimal adsorption temperature was 75 °C and the optimal adsorption time was 40 min. A total of 36 volatile aroma compounds were identified by GC–MS, including 8 aldehydes, 2 ketones, 4 alcohols, 15 alkenes, and 4 alkanes. Hexanal, 3-Octanone, 2-Undecanone, (*E*)-Nerolidol, and (*Z*)-β-Farnesene made great aromatic contributions. Among them, Hexanal, 3-Octanone, 2-Undecanone were the key aroma compounds for which odor activity values (OAVs) were more than 1. (*E*)-Nerolidol showed odor modification in the forest samples and showed a key aroma effect in greenhouse samples. (*Z*)-β-Farnesene showed odor modification in greenhouse samples. 3-Octanone was the largest contributing compound for which the OAV was more than 60. The total content of volatile aroma compounds first increased and then decreased with growth time; it reached the highest level at 48 h: 2203.7 ± 115.2 μg/kg for the forest environment and 4516.6 ± 228.5 μg/kg for the greenhouse environment. The aroma was the most abundant at this time. All samples opened their umbrella at 84 h and become inedible. Principal component analysis (PCA), hierarchical cluster analysis (HCA), and orthogonal partial least squares discriminant analysis (OPLS–DA) were combined to analyze the aroma difference of *S. rugosoannulata* under two cultivation modes. PCA and HCA could effectively distinguish the aroma difference in different growth stages. Under different culturing methods, the aroma substances and their changes were different. The samples were divided into two groups for forest cultivation, while the samples were divided into three groups for greenhouse cultivation. At the end of growth, the aroma of *S. rugosoannulata* with the two cultivation modes was very similar. OPLS–DA clearly distinguished the differences between the two cultivation methods; 17 key aroma difference factors with variable importance projection (VIP) > 1 were obtained from SPLS–DA analysis.

## 1. Introduction

*Stropharia rugosoannulata* is a deciduous mushroom that belongs to the Strophariaceae family and is one of the top 10 mushrooms on the market [1]. It grows naturally in grassland or forest margins and is widely distributed in Asia, Europe, and America [1,2]. *Stropharia rugosoannulata* has a good taste, is popular, and has a high yield in China. It is rich in polysaccharides, flavonoids, saponins, vitamins, lectin, peptides, and steroid compounds [2,3,4,5,6,7,8]. Extracts of the edible mushroom have antibacterial capacity and antioxidant activity [9,10]. Zhang et al. showed that polysaccharides of *Stropharia rugosoannulata* have inhibitory activity on the proliferation of Hep G2 and L1210 cells and inhibited the activity of HIV-1 reverse transcriptase [5]. *Stropharia rugosoannulata* was found to possess some novel umami-active peptides [6]. The key active amino acid sites of the peptide binding to the T1R1/T1R3 receptor were studied [7]. Four steroid compounds were isolated from the fruiting body which can regulate plant growth [8].

At present, there are many cultivation modes of *Pleurotus giganteus*, including field cultivation, forest cultivation, and greenhouse cultivation [11]. Although greenhouse cultivation can control the growing environment and the yield is good, the investment is large and the cost is high [11]. Field cultivation and forest cultivation costs are low; forest cultivation does not occupy farmland resources and is more respected by the government [4,11]. At the same time, some studies have reported that there may be negative effects of greenhouse cultivation [12,13]. Xue et al. reported that long-term greenhouse cultivation increased soil salinity, soil total C and N contents, extractable organic C and extractable organic N contents, and N_2_O emission; it significantly affected soil microbial community composition [12]. Zhou and Wu’Wu’s study showed that greenhouse cultivation decreased the alpha diversity and spatial turnover rate of the soil fungal community [13]. The cultivation of *Stropharia rugosoannulata* under the forest may provide a positive interaction effect with the forest. It would increase soil nutrients, improve soil fertility, optimize microbial communities, and increase the number of soil-beneficial microorganims [14]. Compared to greenhouse cultivation, *S. rugosoannulata* under the forest had higher contents of protein, calcium, polysaccharides, selenium, nicotinic acid, folic acid, and triterpenoids and its aqueous extract and ethanolic extract had stronger antibacterial and antioxidant activities [4].

The volatile aroma compounds of *S. rugosoannulata* include aldehydes, alcohols, acids, esters, ketones, and other volatile organic compounds. Lu et al. identified 50 volatile aroma compounds from *S. rugosoannulata*; alcohols, esters, alkanes, and ketones were the main compounds detected [15]. Bao et al. identified 87 volatile compounds of *S. rugosoannulata*, including 13 aldehydes, 17 esters, 18 alcohols, 4 alkanes, 9 ketones, 8 acids, and 4 pyrazines [16]. Volatile aroma compounds are an important component of food flavor. People’s food preferences mainly depend on its flavor, including volatile aroma components. Therefore, the study of volatile aroma components is important. Wei et al. evaluated the difference in nutritional ingredients, biological activities, and pharmacological activities of *S. rugosoannulata* under greenhouse and forest conditions [4]. But there is no report about the difference in its volatile aroma components under the two cultivating environments. The aroma component and its contents in edible fungi depend on factors such as the cultivation environment, growth and development stage, and culture medium. It is necessary to study the effect of the two growth environments on its flavor.

In this study, the volatile aroma components of *S. rugosoannulata* under different cultivation environments were determined by a headspace solid phase micro extractions–gas chromatograph–mass spectrometer (SPME-GC-MS). The aroma differences were analyzed from growth to storage. Principal component analysis was used to compare the aroma differences in *S. rugosoannulata* under different growth stages of the same cultivation mode and also to compare the aroma differences in *S. rugosoannulata* under different cultivation modes. The study will provide support for the optimization of cultivating, picking, and storing for *S. rugosoannulata*.

## 2. Materials and Methods

### 2.1. Materials

*S. rugosoannulata* was obtained from the Hangzhou Academy of Agricultural Sciences, its name was Jinqiu 18, and the main cultivation substrate was 70.0% rice husk and 30.0% wood chips. It was cultivated under the moso bamboo (Phyllostachys edulis) in the Baiyan Temple forest Farm in Hangzhou. Altitude: 50 m, slope: 15–22°. The district has a warm, temperate, and continental monsoon climate characterized by hot and rainy summers and cold and dry winters. It has an annual mean temperature of 16.3 °C, 1467 h of sunshine, and 134 days of rain, with precipitation being 1460 mm. The average relative humidity is 78.3%. Another strain was cultivated in a greenhouse, with the temperature being 22–28 °C and the average relative humidity 75.0–90.0%.

Hexanal and 1-Hexanol (Analytical standard, ≥98.0%) were purchased from Tokyo Chemical Industry (Shanghai) Co., Ltd. (Shanghai, China). (*E*)-Nerolidol (Analytical standard, ≥98.0%) was purchased from Shanghai Aladdin Biochemical Technology Co., Ltd. (Shanghai, China). 3-Octanone, (*Z*)-β-Farnesene, and 2-Undecanone (Analytical standard, ≥98.0%) were purchased from Shanghai Acmec Biochemical Co., Ltd. (Shanghai, China). Methanol (HPLC) and Ethanol (HPLC) were from Tedia Company, Inc. (Hangzhou, China).

### 2.2. Sample Preparation

The samples were picked and put into a clean PE food bag, placed into a 4 °C incubator, and then sent to the laboratory within 2 h. The soil and other stains on the surface were washed with pure water and the water was dried with absorbent paper. The samples were cut into 0.4–0.6 mm cubes and the 4.0 g samples were weighed and put into a 15-mL headspace bottle for SPME-GC-MS analysis.

The growth time was defined as 0 h when the *S. rugosoannulata* had just grown out of the surface. The samples were collected at 12, 24, 36, 48, 60, 72, and 84 h to study the changes in volatility during the growth stage. The samples at the 48 h growth stage were studied to optimize the adsorption conditions of SPME.

### 2.3. Optimization of the Adsorption Conditions of SPME

Samples weighing 4.0 g were weighed and put into a 15-mL headspace bottle. A pretreated SPME fiber (65 μm DVB/CAR/PDMS, Supelco, Bellefonte, PA, USA) was introduced into the vial headspace to absorb the volatiles.

The adsorption temperatures were selected as 30 °C, 45 °C, 60 °C, 75 °C, and 90 °C, with treatment lasting for 30 min while the effect of the adsorption temperature on the volatile components was investigated. After the optimal adsorption temperature was obtained, the adsorption times of 10 min, 20 min, 30 min, 40 min, and 50 min were selected to further investigate the effect of the adsorption time on the volatile components.

### 2.4. Determination of Volatile Aroma Compounds via SPME–GC–MS

Volatile compounds were extracted via the headspace solid-phase microextraction (SPME) method and the extracts were detected via a gas chromatography/mass spectrometry (7890A/5975C, GC/MS) system (Agilent Technologies, Inc., Santa Clara, CA, USA).

GC: The chromatographic column used was the DB-5 capillary column (30 m × 0.25 mm × 0.25 µm, Agilent J&W, Santa Clara, CA, USA). The start temperature of the column was set to 50 °C and kept for 1 min; then, the temperature was increased to 260 °C and maintained for 3 min at a rate of 5 °C/min. The inlet temperature was 250 °C. The carrier gas was nitrogen (≥99.9%) and the flow rate of the carrier gas was 1.0 mL/min. The desorption time was 2 min. There was no split.

MS: The energy of the electron impact ionization was set to 70 eV; the temperature of the transfer line and ion source was set to 230 °C and 280 °C, respectively. Solvent delay was set at 0 s using the full scan mode. The mass scan ranged from 15 to 550 *m*/*z*.

Identification and quantification of volatile compounds: Identification was accomplished by matching mass spectra and odor properties to those recorded in the NIST 14.0 Library (https://www.nist.gov/nist-researchlibrary (accessed on 20 July 2022)) and the AromaChem library (https://www.alpha-mos.com/ (accessed on 13 September 2022)).

Standard sample preparation: Hexanal, 3-Octanone, 1-Hexanol, (*E*)-Nerolidol, (*Z*)-β-Farnesene, and 2-Undecanone solutions with different concentrations were prepared for GC–MS analysis. We took Hexanal as the external standard for aldehyde, 3-Octanone as the external standard for ketone, (*Z*)-β-Farnesene as the external standard for terpenes, and (*E*)-Nerolidol as the external standard for alkanes and other compounds.

The relative content of the other volatile aroma compounds was quantified based on the ratio of its peak area relative to the peak area and concentration of the external standard. The formula of the relative content of each volatile compound was specified as:

(1) $$C_{i}=\frac{x}{\omega \times 4}\times \omega_{i}\times 1000$$

where “4” means “4.0 g samples”, indicative of the quality of the sample, and “1000” is the coefficient converted from “g” to “kg”.

$C_{i}$—Volatile compound’s relative content in the sample, μg/kg;
x—The external standard content in the sample, μg;
ω—The peak area of the external standard, %;
$\omega_{i}$—The peak area of the target volatile compound, %;

### 2.5. Odor Activity Values for Hexanal, 3-Octanone, (E)-Nerolidol, (Z)-β-Farnesene, and 1-Hexanol in S. rugosoannulata

Odor activity values (OAVs) were applied to evaluate the aroma contributions of the compounds in *S. rugosoannulata.* The OAV of the volatile aroma compound was specified as follows [17]:

(2) $${OAV}_{i}=\frac{C_{i}}{T_{i}}$$

where

${OAV}_{i}$—The odor activity values of the volatile compound (*i*);
$C_{i}$—The content of the volatile compound (*i*) in the sample, μg/kg;
$T_{i}$—The odor threshold of the volatile compound (*i*), μg/kg;

### 2.6. Statistical Analysis

All experiments were conducted in triplicate and the results were expressed as mean ± standard deviation. The data statistics were obtained using WPS 2019 (Beijing Kingsoft Office Software, Inc. Beijing, China). Significance analysis, Pearson analysis, and principal component analysis (PCA) were performed using SPSS 20.0 (SPSS Inc., Chicago, IL, USA). Graphics were produced using Origin 9.5 (Originlab, Northampton, MA, USA). Hierarchical cluster analysis (HCA) and orthogonal partial least squares discriminant analysis (OPLS–DA) were conducted at the following web link (https://cloud.metware.cn/#/tools/tool-list (accessed on 20 May 2023)).

## 3. Results and Discussion

### 3.1. Effect of the Adsorption Temperature on Volatile Components of S. rugosoannulata

**Figure 1:**
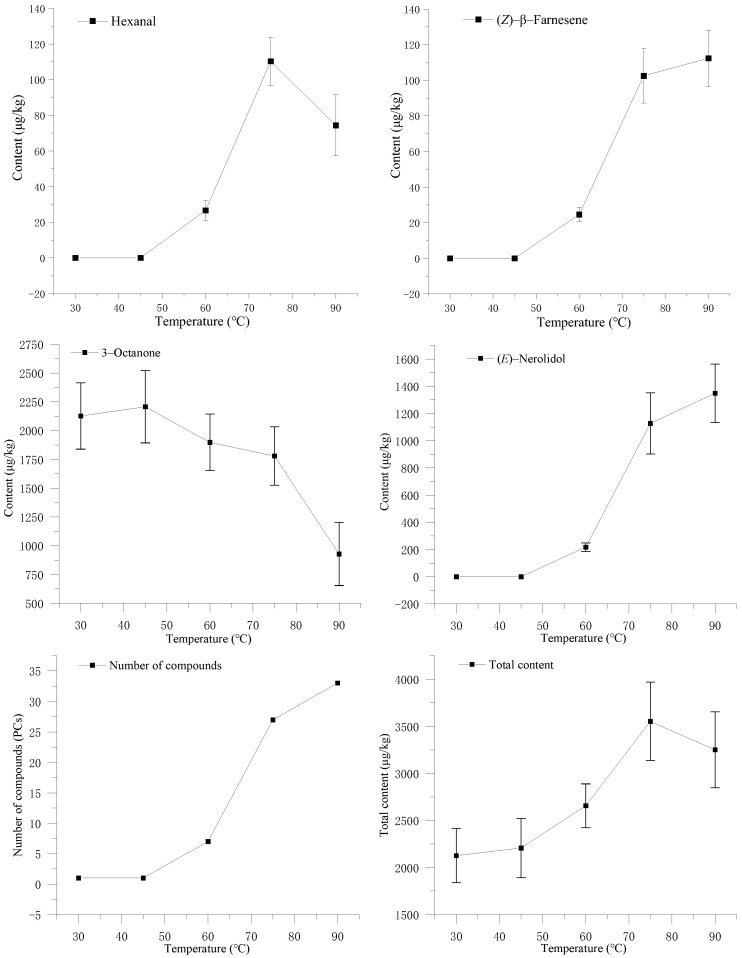
Effects of the adsorption temperature on the volatile components of *S. rugosoannulata*.

Figure 1 shows the adsorption change of Hexanal, 3-Octanone, (*E*)-Nerolidol, and (*Z*)-β-Farnesene at 30 °C, 45 °C, 60 °C, 75 °C, and 90 °C, as well as the adsorption of the total volatiles (TVs). From Figure 1, the adsorption capacity of Hexanal, (*E*)-Nerolidol, (*Z*)-β-Farnesene, and the total volatile compounds was positively correlated with temperature, while the adsorption capacity of 3-octanone was negatively correlated with temperature. When the temperature was 30 °C and 45 °C, only 3-octanone was detected. 3-octanone is a natural fragrance with characteristic mushroom and cheese aromas [18] which are common aromas of edible mushrooms [19]. Hexanal, (*E*)-Nerolidol and (*Z*)-β-Farnesene are also common in food, and they are the four aromas with the higher content of volatile components in the mushroom. At the adsorption temperature of 90 °C, the number of volatile compounds reached the maximum and the total adsorption capacity decreased. Compared with 75 °C, the alkanes and fatty acid methyl esters increased at 90 °C and the high temperature may lead to aroma decomposition and esterification. Considering the number of adsorption compounds and the adsorption capacity of the main compounds, 75 °C was selected as the optimal adsorption temperature.

### 3.2. Effect of Adsorption Time on Volatile Components of S. rugosoannulata

**Figure 2:**
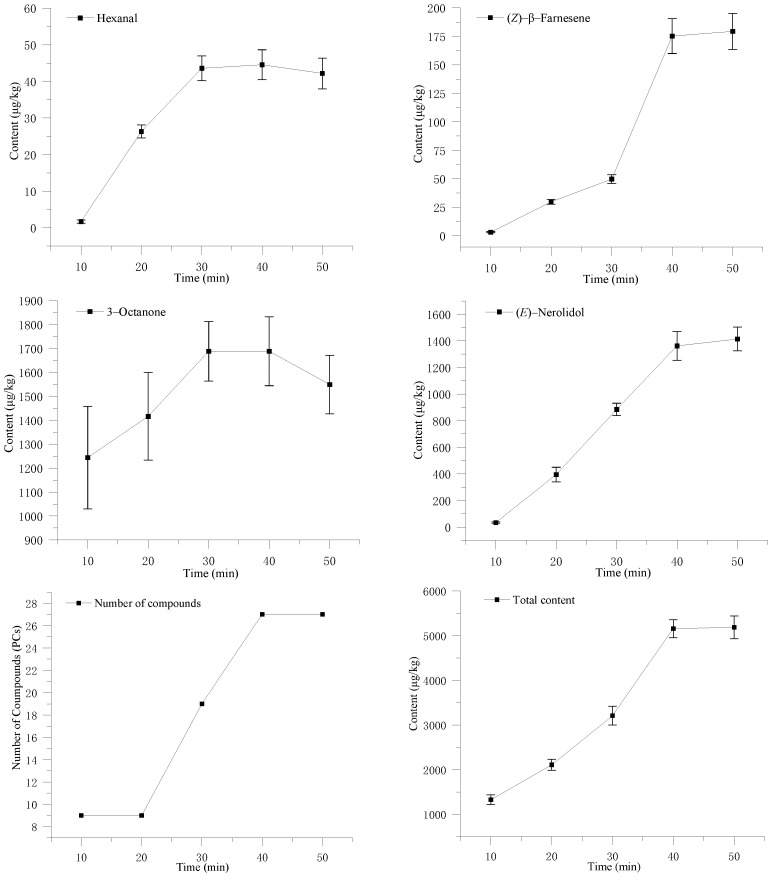
Effects of adsorption time on the volatile components of *S. rugosoannulata*.

Figure 2 shows the adsorption change of Hexanal, 3-Octanone, (*E*)-Nerolidol, and (*Z*)-β-Farnesene at 10 min, 20 min, 30 min, 40 min, and 50 min under 75 °C, as well as the adsorption of the total volatiles (TVs). From Figure 2, the adsorption capacity of Hexanal, 3-octanone, (*E*)-Nerolidol, and (*Z*)-β-Farnesene as well as the total volatile compounds were positively correlated with time. The Pearson index of Hexanal, (*E*)-Nerolidol, and (*Z*)-β-Farnesene was, respectively, 0.94, 0.99, and 0.95, while for 3-Octanone it was only 0.51. 3-Octanone was the main volatile obtained via short-time adsorption at a low temperature. With the increase in the adsorption temperature and adsorption time, the volatiles adsorbed are more abundant, meaning the total adsorption increases. When the adsorption time exceeds 30 min, the adsorption rate of Hexanal and 3-Octanone tends to be balanced and (*E*)-Nerolidol and (*Z*)-β-Farnesene continue to rise. When the adsorption time exceeds 40 min, the adsorption of (*E*)-Nerolidol and (*Z*)-β-Farnesene tends to be balanced and the increases in the total adsorption capacity of the volatiles have the same trend. The adsorption time was selected as 40 min.

### 3.3. Volatile Substances Changes in S. rugosoannulata during Growth under a Forest and Greenhouse

There were 36 signals identified in the *S. rugosoannulata.* A total of 22 signals were identified in the *S. rugosoannulata* under the forest (Table 1), including 8 aldehydes, 2 ketones, 2 alcohols, 4 terpenes, 4 alkanes, and 2 other substances. Whereas, 34 signals were identified under the greenhouse (Table 2), including 6 aldehydes, 2 ketones, 4 alcohols, 15 terpenes, 4 alkanes, and 3 other substances. (*E*,*E*)-2,4-Decadienal and (*E*,*E*)-2,4-Decadienal were detected in forest environment products, but not in greenhouse products. Whereas, Globulol, Cedrol, β-Bisabolene, Calamenene, δ-Cadinene, α-Cubebene, α-Curcumene, α-Bergamotene, 1,2,3,5,6,7,8,8a-Octahydro-1-methyl-6-methylene-4-(1-methylethyl)naphthalene, α-Muurolene, Copaene, Seychellene, Cedrene, and 2,3-Dimethylnaphthalene were detected in greenhouse products, but not in forest products. In total, 20 aroma compounds were all presented in *S. rugosoannulata* both under the forest and under the greenhouse; they were Hexanal, Benzaldehyde, (*E*)-2-Octenal, Benzeneacetaldehyde, Nonanal, Decanal, 3-Octanone, 2-Undecanone, (*E*)-Nerolidol, 1-Hexanol, (*Z*)-β-Farnesene, Thujopsene, α-Cubebene, β-Elemene, Methoxy-phenyl-oxime, 2-Methyl-naphthalene, Tetradecane, Pentadecane, Hexadecane, and Heptadecane. Under the greenhouse, Decanal, Benzeneacetaldehyde, and (*E*)-2-Octenal appeared during growth; Decanal was detected first at 24 h, Benzeneacetaldehyde was detected first at 48 h, and (*E*)-2-Octenal was detected first at 72 h. All of the three above compounds presented at all detection times for *S. rugosoannulata* in the forest. Under the greenhouse, Globulol, Cedrol, β-Cubebene, 1,2,3,5,6,7,8,8a-Octahydro-1-methyl-6-methylene-4-(1-methylethyl)naphthal, Copaene, Seychellene, Cedrene, and β-Elemene were only detected at 12 h and then disappeared with growth time.

**Figure 3:**
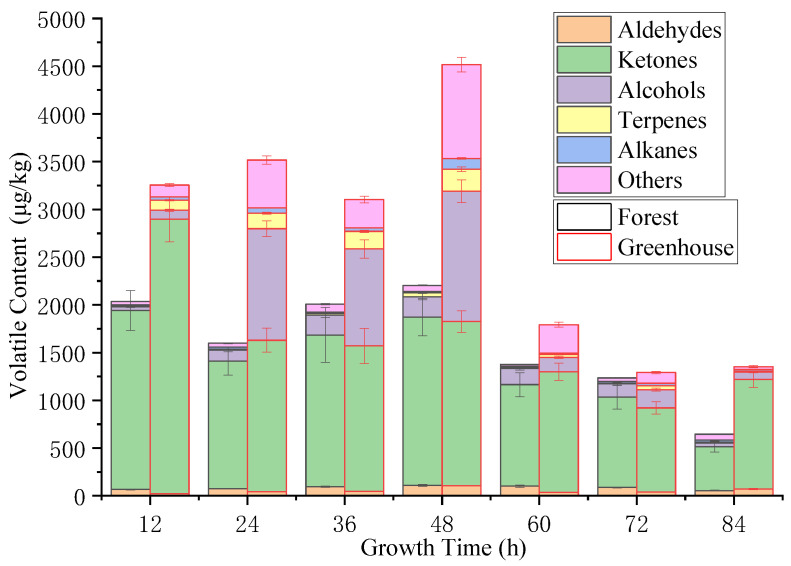
Changes in content for each category of volatile aroma compound in *S. rugosoannulata* during growth.

The changes in content for each category of volatile aroma compound in *S. rugosoannulata* are shown in Figure 3. Combined with Table 1 and Table 2, it confirmed that ketones, alcohols, and aldehydes were the main volatile aromas in *S. rugosoannulata* and that they were mainly C6–C15 compounds. The alcohols had pleasant woody and fragrant aromas, aldehydes had a low threshold value and fatty aromas, and the ketones had floral and fruity aromas, which contributed significantly to the overall flavor of the *S. rugosoannulata* [17,20]. No matter if it was cultivated under a forest or under a greenhouse, the content of the ketones in *S. rugosoannulata* was always the highest (Figure 3). The total content of the volatiles’ aroma compounds first increased and then decreased with the growth time for *S. rugosoannulata* under greenhouse or forest cultivation. Its content reached its highest at 48 h, when the harvest aroma was the most abundant. At 84 h, the volatile components decreased to their lowest content; at this point, the mushrooms had all opened in such a way as to become umbrella-like, with the edible flavor of the compounds having turned bad. The quantity and content of the volatile compounds of the samples in the greenhouse were higher than those in the forest. The difference was significant (*p* < 0.01) at the early growth stage (12–48 h). Their content difference reached the largest at 48 h and the content of the former was 2.14 times that of the latter. At the later growth stage (60–84 h), the volatile components decreased sharply with time for two cultivation modes and their content had no significant difference (*p* > 0.1). The difference in the content of the volatile components under two cultivation modes may be related to the growing environment. Studies have shown that rainfall, air humidity, soil moisture, and atmospheric pressure are significantly related to the quality and the yield of *S. rugosoannulata* [21]. The stable environment with sufficient moisture in the greenhouse conditions is more conducive to the growth of *S.rugosoannulata* as it is good for promoting flavor formation for *S. rugosoannulata*. Moreover, the slow air circulation in greenhouses results in less loss of volatile components, while the situation is the opposite in a wild environment. This may be one of the reasons for the difference in the quantity and content of the volatile components of *S. rugosoannulata* under the two cultivation modes.

Two ketones were detected, including 3-Octanone and 2-Undecanone. 3-Octanone has earthy and mushroom notes and 2-Undecanone has milky and fruity notes [17]. For samples in the forest, the content of 3-Octanone was 1855.1 ± 208.7 μg/kg, accounting for 91.2% of the total volatiles at 12 h, and the content of 3-Octanone was 1732.1 ± 192.7 μg/kg, accounting for 78.6% of the total volatiles at 48 h. For samples under the greenhouse, the content of 3-Octanone was 2861.0 ± 234.7 μg/kg, accounting for 87.9% of the total volatiles at 12 h, and the content of 3-Octanone was 1688.4 ± 108.8 μg/kg, accounting for 37.4% of the total volatiles at 48 h. From Table 3, the OAV values of 3-Octanone under the under-forest cultivation and greenhouse cultivations methods are both more than 60.0, showing that 3-Octanone has the greatest contribution to the flavor of *S. rugosoannulata.* 2-Undecanone first increased and then decreased with the growth time, with its content reaching its highest values at 48 h, with 30.4 ± 2.3 μg/kg for the forest conditions and 31.7 ± 4.7 μg/kg for the greenhouse conditions. The OAV values of 2-Undecanone are more than 4.3 (Table 3), showing that it also has a large role in aroma contribution. (*E*)-Nerolidol has a fragrant, floral, and fruity aroma [22]. It ranked second place in terms of the content of the volatile compounds of *S. rugosoannulata* (Table 1 and Table 2). Its content increased and then reached its highest value at 48 h, which was 12.7 ± 32.4 μg/kg for the forest conditions and accounting for 9.7%, and 1362.6 ± 188.7 μg/kg for the greenhouse conditions and accounting for 30.2%; after that, its content decreased with time. The content of (*E*)-Nerolidol in the forest samples is six times lower than that in the greenhouse samples. The aroma effect of this component is relatively lower in the forest products but more obvious in greenhouse products. Wei et.al [10] had reported that (*E*)-Nerolidol was present in essential oils from fresh and dried *S. rugosoannulata.* The content of 3-Octanone and (*E*)-Nerolidol in the forest samples was lower than that in the greenhouse samples, with the difference being more than 1000 μg/kg in samples detected at the 12 h point. This situation may be caused by the stable environment found in greenhouses. 1-Hexanol has a floral and fatty aroma [17] but may be a sign of corruption for *S. rugosoannulata.* At the growth stage of 12–60 h, its content was very low: less than or equal to 1.0 ± 0.1 μg/kg in the forest samples and 3.4 ± 0.3 μg/kg in the greenhouse samples. At the growth stage of 72–84 h, its content rose rapidly, with the largest content being 49.8 ± 7.3 μg/kg in the forest samples and 63.9 ± 6.5 μg/kg in the greenhouse samples, with this latter value having increased 30–48 times. Hexanal, Benzaldehyde, and Nonanal had a relatively higher content in the two cultivation modes compared with other aldehydes. Hexanal had the highest concentration, showcasing a grassy and apple fragrance aroma [17,22]. The OAV values of Hexanal were more than 8.6 and it ranked second place for aroma contribution in key volatile compounds of *S. rugosoannulata* (Table 3). (*Z*)-β-Farnesene is a major component of several plant essential oils with a slight balm aroma [23], mainly existing in rose oil, orange oil, and other essential oils. It is also one of the raw materials for the synthesis of vitamin E [24]. Li et Al. had detected (*Z*)-β-Farnesene in the pleurotus eryngii [25]. The (*Z*)-β-Farnesene OAV values of samples under the forest and greenhouse differed greatly: 0.0 for the forest samples and 0.3 for the greenhouse samples. Studies had shown that compounds with an OAV range of 0.1–1.0 can modify the aroma and that a value greater than 1.0 indicates that the compound is a key aroma substance; indeed, the greater the value, the greater the contribution to the overall odor characteristics of the sample [26]. Therefore, (*Z*)-β-Farnesene had a certain aroma modification effect upon the greenhouse samples. In conclusion, for forest samples, the key aroma substances of *S. rugosoannulata* are Hexanal, 3-Octanone, and 2-Undecanone and the modification aroma substance is (*E*)-Nerolidol; for the greenhouse samples, the key aroma substances of *S. rugosoannulata* are Hexanal, 3-Octanone, 2-Undecanone, and (*E*)-Nerolidol and the modification aroma substance is (*Z*)-β-Farnesene.

### 3.4. Aroma Difference Analysis of S. rugosoannulata

Data analysis mainly includes unsupervised analysis and supervised analysis. Unsupervised analysis can cluster samples in the absence of unknown sample information, such as via PCA and HCA. Unsupervised analysis methods cannot ignore intra-group errors, while supervised analysis methods can reduce intra-group random errors and highlight systematic errors between each group via partial least squares-discriminant analysis (PLS-DA) and OPLS-DA. PCA, HCA, and OPLS-DA will be combined to discuss the aroma difference of *S. rugosoannulata* here.

**Figure 4:**
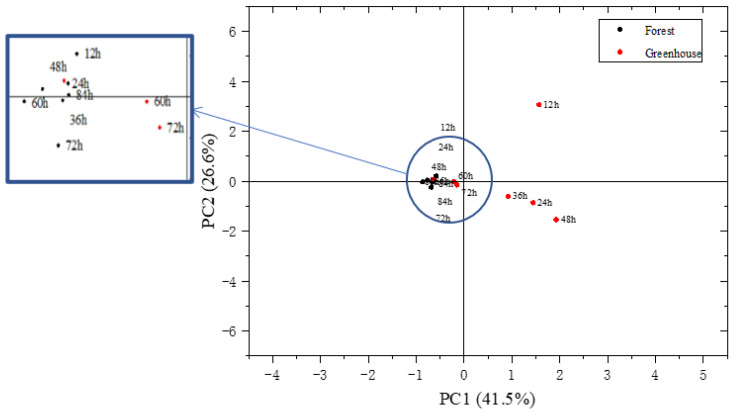
PCA analysis of the characteristic aroma compounds presented in the *S. rugosoannulata*.

A PCA based on 36 aromas detected via GC-MS is shown in Figure 4. It expresses the difference in the aroma characteristics of *S. rugosoannulata* at different growth stages more directly. A total of five principal components with eigenvalues greater than 1 were extracted and the cumulative variance contribution rate was 91.4%. Figure 4 shows the scores of the first two principal components; the contribution rate of PC1 was 41.5% while the contribution rate of PC2 was 26.6%. The cumulative variance contribution rate was 68.1%. PC1 and PC2 contained a large amount of information on the samples and could reflect the characteristic aromas for *S. rugosoannulata.* The distance between samples in Figure 4 represents the aroma difference. From Figure 4, it shows that the aromas of the forest samples at each growth stage were relatively similar, while the aromas of greenhouse samples were greatly different. Furthermore, the greenhouse samples at 24–48 h have a similar aroma, and the greenhouse samples at 60–72 h also have a similar aroma.

**Figure 5:**
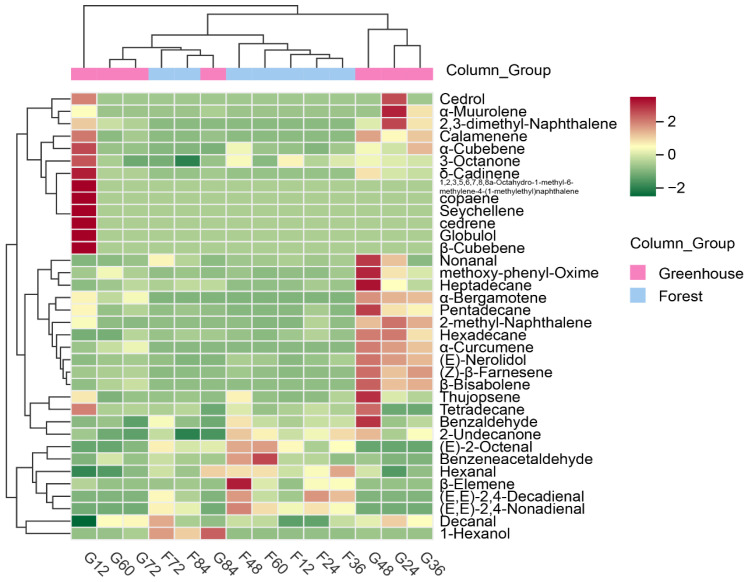
HCA heat map of volatile aroma compounds in *S. rugosoannulata* during growth. (G_12–84_ samples at 12–84 h for the greenhouse; F_12–84_: samples at 12–84 h for the forest).

Figure 5 shows the HCA heat map of the volatile aroma compounds in *S. rugosoannulata* during growth. It shows that the forest samples at 12–60 h can be regarded as one group and the forest samples at 72–84 h can be regarded as the other group. The greenhouse samples had relatively large aroma differences. Samples at 12 h can be taken as a single group, samples at 24–48 h can be taken as one group, and samples at 60–84 h can be taken as another group. There is a large difference between the two cultivation methods at the early stage of growth, while at the end of growth (especially at the 72 h and 84 h stages) the samples under the two forms of cultivation are very similar.

**Figure 6:**
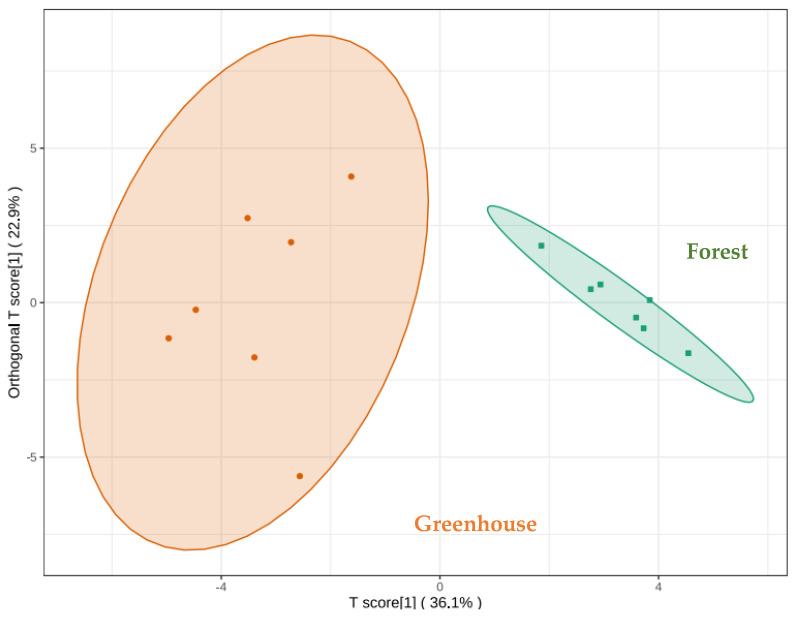
Score chart of OPLS−DA model of volatile aroma compounds in *S. rugosoannulata*.

Figure 6 shows the score chart of the OPLS–DA mode, with R^2^X = 0.59, R^2^Y = 0.93, and Q^2^ = 0.83 indicating that the model is stable and reliable. There was a significant difference between the two cultivation methods and the 95.0% confidence interval was obviously divided into two blocks. It indicates that there were significant differences in volatile aroma substances between those under greenhouse cultivation and those under the forest cultivation environment. With variable importance projection (VIP) > 1 as the selecting criterion, the main potential component causing aroma differences between the two cultivation methods could be obtained. The larger the VIP value, the greater the influence of this component on aroma differences. The results show that there are 17 key factors that influence aroma differences between the two cultivation modes, which are (*E*,*E*)-2,4-Nonadienal (VIP = 1.6), (*E*,*E*)-2,4-Decadienal (VIP = 1.6), δ-Cadinene (VIP = 1.6), α-Bergamotene (VIP = 1.5), 2-Methyl-naphthalene (VIP = 1.5), α-Curcumene (VIP = 1.5), (*E*)-2-Octenal (VIP = 1.4), Calamenene (VIP = 1.4), β-Bisabolene (VIP = 1.4), Pentadecane (VIP = 1.3), (*Z*)-β-Farnesene (VIP = 1.3), β-Elemene (VIP = 1.3), Methoxy-phenyl-oxime (VIP = 1.2), Benzeneacetaldehyde (VIP = 1.2), Hexanal (VIP = 1.1), α-Muurolene (VIP = 1.1), and 2-Methyl-naphthalene (VIP = 1.1).

## 4. Conclusions

In order to analyze the aromas of *S. rugosoannulata*, the effect of adsorption temperature and time on the volatile components of *S. rugosoannulata* via GC-MS has been studied. The optimal adsorption temperature was selected as 75 °C and the optimal time was selected as 40 min.

A total of 36 aroma compounds were identified in the studied *S. rugosoannulata.* For forest cultivation, 22 aroma compounds were identified, including 8 aldehydes, 2 ketones, 2 alcohols, 4 terpenes, 4 alkanes, and 2 other substances; for greenhouse cultivation, 34 aroma compounds were identified, including 6 aldehydes, 2 ketones, 4 alcohols, 15 terpenes, 4 alkanes, and 3 other substances. (*E*,*E*)-2,4-Decadienal and (*E*,*E*)-2,4-Decadienal were unique to the forest samples; while Globulol, Cedrol, β-Bisabolene, Calamenene, δ-Cadinene, α-Cubebene, α-Curcumene, α-Bergamotene, 1,2,3,5,6,7,8,8a-Octahydro-1-methyl-6-methylene-4-(1-methylethyl)naphthalene, α-Muurolene, Copaene, Seychellene, Cedrene, and 2,3-Dimethylnaphthalene were unique to the greenhouse samples. There were 20 identical ingredients present in both cultivation methods. Among them, for forest samples, the key aroma substances of *S. rugosoannulata* were Hexanal, 3-Octanone, and 2-Undecanone and the modification aroma substance was (*E*)-Nerolidol; for the greenhouse samples, the key aroma substances of *S. rugosoannulata* were Hexanal, 3-Octanone, 2-Undecanone, and (*E*)-Nerolidol and the modification aroma substance was (*Z*)-β-Farnesene. The total content of the volatiles’ aromas reached the highest at 48 h, when the aroma was the most abundant. At 84 h, the volatile components decreased to their lowest value and were all opened in an umbrella-like manner with the edible flavor turning bad.

PCA, HCA, and OPLS−DA were combined to discuss the aroma difference of *S. rugosoannulata.* The PCA and HCA could clearly distinguish the aroma difference at different growth stages. For forest cultivation, the aroma of *S. rugosoannulata* at the 12–60 h growth stage is similar and the aroma of *S. rugosoannulata* at the 72–84 h growth stage is similar. For greenhouse cultivation, the aroma of *S. rugosoannulata* at the 24–48 h growth stage is similar and the aroma of *S. rugosoannulata* at the 60–84 h growth stage is similar. The aromas of *S. rugosoannulata* at 12 h were quite different from those mentioned above. At the end of growth, the aromas of *S. rugosoannulata* under two cultivation modes were very similar. OPLS−DA can more clearly distinguish the differences between the two cultivation methods; 17 key aroma difference factors were obtained.

Greenhouse conditions are more standard and stable compared with forest conditions. Furthermore, forest conditions have different types. Therefore, it will be necessary to study more forest types in the future.

## Figures and Tables

**Table 1:** Change in the relative content of volatile aromas compounds in the *S. rugosoannulata* under the forest during growth by GC–MS.

Compound		CAS#	Formula	Relative Content (μg/kg)						Forest
				12 h	24 h	36 h	48 h	60 h	72 h	84 h
Aldehyde	Hexanal	66-25-1	C_6_H_12_O	37.8 ± 3.3 ^c^	45.4 ± 3.1 ^b^	61.4 ± 4.6 ^a^	51.7 ± 5.6 ^ab^	51.8 ± 4.5 ^ab^	38.1 ± 2.0 ^c^	30.1 ± 2.3 ^d^
	Benzaldehyde	100-52-7	C_7_H_6_O	8.5 ± 2.5 ^cd^	11.5 ± 2.8 ^bc^	11.6 ± 1.9 ^bc^	20.0 ± 2.8 ^a^	11.3 ± 3.6 ^bc^	16.2 ± 2.5 ^ab^	5.9 ± 1.3 ^d^
	(*E*)-2-Octenal	2548-87-0	C_8_H_14_O	8.0 ± 0.9 ^b^	3.4 ± 0.3 ^d^	7.6 ± 0.6 ^b^	12.1 ± 1.4 ^a^	12.7 ± 1.3 ^a^	8.5 ± 0.7 ^b^	5.5 ± 0. 5 ^c^
	Benzeneacetaldehyde	122-78-1	C_8_H_8_O	2.7 ± 0.4 ^c^	2.6 ± 0.7 ^c^	0.8 ± 0.2 ^e^	10.6 ± 1.5 ^b^	14.6 ± 0.8 ^a^	3.4 ± 0.9 ^c^	1.9 ± 0.3 ^d^
	Nonanal	124-19-6	C_9_H_18_O	6.4 ± 1.7 ^b^	5.7 ± 1.2 ^b^	6.7 ± 1.3 ^b^	5.6 ± 1.1 ^b^	5.8 ± 1.0 ^b^	12.0 ± 1.1 ^a^	7.0 ± 0.9 ^b^
	Decanal	112-31-2	C_10_H_20_O	1.5 ± 0.4 ^c^	1.6 ± 0.3 ^c^	3.2 ± 0.4 ^b^	3.3 ± 0.4 ^b^	3.1 ± 0.5 ^b^	5.5 ± 0.9 ^a^	2.8 ± 0.3 ^b^
	(*E*,*E*)-2,4-Decadienal	25152-84-5	C_10_H_16_O	0.4 ± 0.4 ^c^	3.2 ± 0.7 ^a^	2.6 ± 0.6 ^ab^	3.1 ± 0.8 ^a^	0.9 ± 0.4 ^c^	1.8 ± 0.4 ^b^	0.6 ± 0.1 ^c^
	(*E*,*E*)-2,4-Nonadienal	5910-87-2	C_9_H_14_O	1.3 ± 0.3 ^b^	1.8 ± 0.3 ^ab^	1.4 ± 0.2 ^b^	2.8 ± 0.6 ^a^	1.8 ± 0.5 ^ab^	1.6 ± 0.3 ^ab^	1.2 ± 0.2 ^b^
Ketone	3-Octanone	106-68-3	C_8_H_16_O	1855.1 ± 208.7 ^a^	1312.2 ± 143.7 ^b^	1560.4 ± 284.3 ^ab^	1732.1 ± 192.7 ^a^	1036.1 ± 122.5 ^bc^	928.4 ± 124.7 ^c^	456.9 ± 55.9 ^d^
	2-Undecanone	112-12-9	C_11_H_22_O	19.6 ± 2.2 ^c^	23.4 ± 3.0 ^bc^	28.4 ± 3.4 ^ab^	30.4 ± 2.3 ^a^	26.2 ± 3.4 ^ab^	19.0 ± 1.8 ^c^	4.1 ± 0.8 ^d^
Alcohols	(*E*)-Nerolidol	7212-44-4	C_15_H_26_O	37.9 ± 6.2 ^h^	113.9 ± 15.3 ^e^	209.7 ± 26.9 ^c^	212.7 ± 32.4 ^c^	169.1 ± 18.6 ^cd^	89.0 ± 10.3 ^f^	-
	1-Hexanol	111-27-3	C_6_H_14_O	0.9 ± 0.1 ^cd^	0.99 ± 0.1 ^c^	1.0 ± 0.1 ^c^	0.8 ± 0.1 ^d^	0.7 ± 0.1 ^d^	49.8 ± 7.3 ^a^	38.0 ± 6.1 ^b^
Terpenes	(*Z*)-β-Farnesene	28973-97-9	C_15_H_24_	2.8 ± 0.4 ^d^	3.7 ± 0.4 ^c^	6.6 ± 0.7 ^b^	14.4 ± 3.1 ^a^	7.4 ± 2.1 ^b^	2.6 ± 0.5 ^d^	3.9 ± 0.8 ^cd^
	β-Elemene	515-13-9	C_15_H_24_	-	2.9 ± 1.1 ^b^	3.0 ± 0.9 ^b^	9.6 ± 1.7 ^a^	2.0 ± 0.9 ^b^	0.2 ± 0.1 ^c^	0.4 ± 0.1 ^c^
	Thujopsene	470-40-6	C_15_H_24_	2.6 ± 0.2 ^d^	2.7 ± 0.2 ^d^	4.8 ± 0.5 ^b^	9.6 ± 1.3 ^a^	3.1 ± 0.2 ^c^	3.2 ± 0.3 ^c^	3.5 ± 0.3 ^c^
	α-Cubebene	17699-14-8	C_15_H_24_	1.4 ± 0.5 ^bc^	0.4 ± 0.6 ^c^	2.1 ± 0.6 ^b^	6.6 ± 1.1 ^a^	1.8 ± 0.5 ^b^	0.5 ± 0.1 ^c^	0.4 ± 0.2 ^c^
Other	Methoxy-phenyl-oxime	1000222-86-6	C_8_H_9_NO_2_	31.8 ± 2.2 ^c^	23.6 ± 2.7 ^d^	77.3 ± 5.1 ^a^	57.6 ± 4.3 ^b^	16.2 ± 1.8 ^e^	31.8 ± 2.9 ^c^	55.1 ± 4.8 ^b^
	2-Methyl-naphthalene	91-57-6	C_11_H_10_	2.1 ± 0.4 ^c^	17.1 ± 1.2 ^a^	5.4 ± 0.9 ^b^	5.4 ± 0.9 ^b^	2.2 ± 0.4 ^c^	5.5 ± 0.7 ^b^	5.5 ± 0.6 ^b^
Alkanes	Tetradecane	629-59-4	C_14_H_30_	3.8 ± 0.2 ^c^	5.3 ± 0.5 ^b^	3.9 ± 0.3 ^c^	6.5 ± 0.7 ^a^	2.5 ± 0.4 ^d^	3.9 ± 0.2 ^c^	4.5 ± 0.6 ^bc^
	Heptadecane	629-78-7	C_17_H_36_	4.4 ± 0.3 ^d^	6.3 ± 0.6 ^b^	5.2 ± 0.4 ^c^	4.1 ± 0.4 ^d^	4.1 ± 0.9 ^cd^	7.0 ± 0.2 ^b^	8.9 ± 1.0 ^a^
	Hexadecane	544-76-3	C_16_H_34_	3.3 ± 0.3 ^b^	5.1 ± 0.6 ^a^	2.7 ± 0.2 ^c^	2.7 ± 0.1 ^c^	1.7 ± 0.5 ^d^	2.8 ± 0.3 ^c^	4.1 ± 0.4 ^ab^
	Pentadecane	629-62-9	C_15_H_32_	2.6 ± 0.2 ^c^	5.5 ± 0.4 ^a^	2.5 ± 0.4 ^c^	2.3 ± 0.3 ^c^	2.3 ± 0.9 ^c^	3.5 ± 0.3 ^b^	4.7 ± 0.9 ^ab^

Note: Different letters in the same row indicate statistically significant differences in the results (*p* < 0.05).

**Table 2:** Change in the relative content of volatile aromas compounds in the *S. rugosoannulata* under the greenhouse during growth by GC–MS.

Compound		CAS#	Formula	Relative Content (μg/kg)						Greenhouse
				12 h	24 h	36 h	48 h	60 h	72 h	84 h
Aldehyde	Hexanal	66-25-1	C_6_H_12_O	12.4 ± 3.1 ^d^	17.6 ± 2.2 ^d^	28.2 ± 3.0 ^c^	38.9 ± 4.2 ^b^	17.5 ± 2.1 ^d^	26.9 ± 3.5 ^c^	54.5 ± 2.0 ^a^
	Benzaldehyde	100-52-7	C_7_H_6_O	4.9 ± 1.7 ^cd^	6.7 ± 1.1 ^bc^	10.2 ± 3.4 ^b^	38.3 ± 11.5 ^a^	6.4 ± 2.3 ^bc^	0.4 ± 0.3 ^e^	2.2 ± 1.1 ^d^
	Nonanal	124-19-6	C_9_H_18_O	3.9 ± 0.7 ^c^	15.1 ± 2.2 ^b^	4.3 ± 0.9 ^c^	23.3 ± 3.9 ^a^	4.5 ± 0.7 ^c^	5.2 ± 0.6 ^c^	4.5 ± 0.9 ^c^
	Decanal	112-31-2	C_10_H_20_O	-	4.9 ± 0.7 ^a^	4.1 ± 0.7 ^a^	3.7 ± 0.7 ^a^	4.2 ± 0.3 ^a^	4.3 ± 1.1 ^a^	2.4 ± 0.6 ^b^
	Benzeneacetaldehyde	122-78-1	C_8_H_8_O	-	-	-	1.5 ± 0.8 ^b^	3.9 ± 1.1 ^a^	1.1 ± 0.6 ^b^	1.7 ± 0.3 ^b^
	(E)-2-Octenal	2548-87-0	C_8_H_14_O	-	-	-	-	-	1.5 ± 0.3 ^b^	6.1 ± 0.5 ^a^
Ketone	3-Octanone	106-68-3	C_8_H_16_O	2861.0 ± 234.7 ^a^	1568.7 ± 124.5 ^bc^	1500.0 ± 181.5 ^bc^	1688.4 ± 108.8 ^b^	1252.0 ± 88.5 ^cd^	872.7 ± 62.5 ^d^	1140.3 ± 83.6 ^d^
	2-Undecanone	112-12-9	C_11_H_22_O	15.5 ± 2.8 ^c^	16.6 ± 1.9 ^c^	24.5 ± 1.7 ^b^	31.7 ± 4.7 ^a^	10.7 ± 2.7 ^d^	9.1 ± 1.3 ^de^	7.1 ± 0.7 ^e^
Alcohols	(E)-Nerolidol	7212-44-4	C_15_H_26_O	62.0 ± 5.8 ^d^	1165.3 ± 83.0 ^ab^	1012.7 ± 125.7 ^b^	1362.6 ± 188.7 ^a^	149.9 ± 21.5 ^c^	185.1 ± 16.8 ^c^	14.5 ± 4.1 ^e^
	1-Hexanol	111-27-3	C_6_H_14_O	1.1 ± 0.2 ^e^	2.1 ± 0.3 ^d^	2.0 ± 0.2 ^d^	3.4 ± 0.3 ^c^	1.8 ± 0.3 ^d^	6.3 ± 0.7 ^b^	63.9 ± 6.5 ^a^
	Globulol	489-41-8	C_15_H_26_O	29.1 ± 5.1 ^a^	-	-	-	-	-	-
	Cedrol	77-53-2	C_15_H_26_O	1.1 ± 0.3 ^a^	1.4 ± 0.2 ^a^	-	-	-	-	-
Terpenes	(Z)-β-Farnesene	28973-97-9	C_15_H_24_	14.8 ± 2.1 ^d^	100.0 ± 11.7 ^b^	122.5 ± 14.1 ^ab^	146.0 ± 22.0 ^a^	20.3 ± 3.3 ^c^	23.9 ± 3.8 ^c^	5.2 ± 1.2 ^e^
	β-Bisabolene	495-61-4	C_15_H_24_	0.1 ± 0.0 ^e^	17.6 ± 1.9 ^b^	19.3 ± 1.6 ^b^	27.2 ± 3.6 ^a^	3.4 ± 0.5 ^d^	5.8 ± 0.8 ^c^	-
	Thujopsene	470-40-6	C_15_H_24_	10.1 ± 0.8 ^b^	6.8 ± 0.8 ^c^	4.8 ± 0.6 ^d^	20.1 ± 3.8 ^a^	1.8 ± 0.3 ^f^	3.2 ± 0.6 ^e^	2.8 ± 0.4 ^e^
	Calamenene	483-77-2	C_15_H_22_	14.0 ± 1.2 ^a^	6.8 ± 0.6 ^c^	9.6 ± 1.3 ^b^	11.7 ± 1.0 ^b^	-	1.4 ± 0.3 ^d^	0.6 ± 0.1 ^e^
	δ—Cadinene	483-76-1	C_15_H_24_	18.5 ± 0.4 ^a^	3.6 ± 0.7 ^c^	2.8 ± 0.7 ^c^	7.5 ± 1.3 ^b^	1.5 ± 0.2 ^d^	1.4 ± 0.3 ^d^	0.8 ± 0.2 ^d^
	α-Cubebene	17699-14-8	C_15_H_24_	17.8 ± 3.0 ^a^	4.8 ± 1.2 ^c^	11.4 ± 1.9 ^b^	6.8 ± 1.3 ^c^	1.6 ± 0.6 ^d^	1.5 ± 0.7 ^d^	-
	α-Curcumene	644-30-4	C_15_H_22_	0.4 ± 0.2 ^e^	5.1 ± 0.8 ^ab^	4.2 ± 0.6 ^b^	5.8 ± 0.4 ^a^	1.5 ± 0.1 ^d^	2.3 ± 0.4 ^c^	-
	α-Bergamotene	17699-05-7	C_15_H_24_	2.4 ± 0.8 ^b^	3.6 ± 0.7 ^ab^	3.4 ± 0.4 ^ab^	4.1 ± 0.6 ^a^	1.1 ± 0.4 ^c^	2.0 ± 0.7 ^bc^	-
	β-Cubebene	13744-15-5	C_15_H_24_	11.29 ± 1.56 ^a^	-	-	-	-	-	-
	1,2,3,5,6,7,8,8a-Octahydro-1-methyl-6-methylene-4-(1-methylethyl)naphthalene	150320-52-8	C_15_H_24_	4.8 ± 1.8 ^a^	-	-	-	-	-	-
	α-Muurolene	31983-22-9	C_15_H_24_	4.5 ± 0.7 ^b^	14.8 ± 1.6 ^a^	5.9 ± 0.8 ^b^	-	-	-	0.7 ± 0.3 ^c^
	Copaene	3856-25-5	C_15_H_24_	2.7 ± 0.5 ^a^	-	-	-	-	-	-
	Seychellene	20085-93-2	C_15_H_24_	1.4 ± 0.2 ^a^	-	-	-	-	-	-
	Cedrene	11028-42-5	C_15_H_24_	1.3 ± 0.5 ^a^	-	-	-	-	-	-
	β-Elemene	515-13-9	C_15_H_24_	0.9 ± 0.2 ^a^	-	-	-	-	-	-
Other	Methoxy-phenyl-oxime	1000222-86-6	C_8_H_9_NO_2_	77.3 ± 10.0 ^f^	401.1 ± 33.3 ^b^	222.2 ± 25.6 ^d^	918.4 ± 70.0 ^a^	289.1 ± 24.7 ^c^	106.3 ± 7.8 ^e^	24.4 ± 6.6 ^g^
	2-Methyl-naphthalene	91-57-6	C_11_H_10_	38.1 ± 3.0 ^c^	83.4 ± 5.9 ^a^	65.6 ± 7.3 ^b^	60.4 ± 7.2 ^b^	5.2 ± 0.7 ^c^	4.0 ± 3.0 ^c^	5.0 ± 4.5 ^c^
	2,3-Dimethylnaphthalene	581-40-8	C_12_H_22_	9.0 ± 2.1 ^b^	15.9 ± 3.7 ^a^	7.5 ± 1.9 ^b^	4.4 ± 0.7 ^c^	3.2 ± 0.6 ^c^	2.0 ± 0.5 ^d^	-
Alkanes	Heptadecane	629-78-7	C_17_H_36_	3.7 ± 0.7 ^d^	15.5 ± 0.0 ^b^	8.7 ± 0.8 ^c^	39.8 ± 3.1 ^a^	5.6 ± 1.2 ^d^	8.6 ± 1.2 ^c^	8.7 ± 0.7 ^c^
	Pentadecane	629-62-9	C_15_H_32_	15.3 ± 1.7 ^b^	17.3 ± 1.7 ^b^	15.5 ± 2.2 ^b^	33.6 ± 2.8 ^a^	3.9 ± 0.5 ^d^	6.8 ± 1.7 ^c^	3.5 ± 0.4 ^d^
	Hexadecane	544-76-3	C_16_H_34_	-	22.0 ± 2.0 ^a^	13.6 ± 1.5 ^b^	21.8 ± 1.8 ^a^	-	5.4 ± 2.7 ^c^	3.7 ± 0.3 ^c^
	Tetradecane	629-59-4	C_14_H_30_	16.1 ± 1.3 ^a^	-	-	17.4 ± 2.2 ^a^	3.9 ± 0.3 ^b^	4.1 ± 1.2 ^b^	-

Note: Different letters in the same row indicate statistically significant differences in the results (*p* < 0.05).

**Table 3:** Comparison of OAVs of mainly volatile compounds in *S. rugosoannulata* during growth.

Main Volatile Compounds	Content (μg/kg)		* Odor Threshold [19] (μg/kg)	OAV	
	Forest	Greenhouse		Forest	Greenhouse
Hexanal	51.7	38.9	4.5	11.5	8.6
3-Octanone	1732.1	1688.4	28.0	61.9	60.3
2-Undecanone	30.4	31.7	7.0	4.3	4.5
1-Hexanol	0.8	3.4	2500.0	0.0	0.0
(E)-Nerolidol	212.7	1362.6	300.0	0.7	4.5
(Z)-β-Farnesene	14.4	146.0	450.0	0.0	0.3

* Note: the odor-threshold testing methods were based on the ATSM29 E679 3-alternative forced choice (3-AFC) method of limits.

## Data Availability

The data that support the findings of this study are available from the corresponding author upon reasonable request.
